# A First *Plasmodium vivax* Natural Infection Induces Increased Activity of the Interferon Gamma-Driven Tryptophan Catabolism Pathway

**DOI:** 10.3389/fmicb.2020.00400

**Published:** 2020-03-17

**Authors:** Rafaella Oliveira dos Santos, Maria Geuziane Soares da Cruz, Stefanie Costa Pinto Lopes, Lucas Barbosa Oliveira, Paulo Afonso Nogueira, Emerson Silva Lima, Irene Silva Soares, Flora Satiko Kano, Andréa Teixeira de Carvalho, Fabio Trindade Maranhão Costa, Christian A. Ganoza, Marcus Vinicius Guimarães de Lacerda, Pritesh Lalwani

**Affiliations:** ^1^Instituto Leônidas e Maria Deane (ILMD), Fiocruz Amazônia, Manaus, Brazil; ^2^Faculdade de Ciências Farmacêuticas, Universidade Federal do Amazonas (UFAM), Manaus, Brazil; ^3^Faculdade de Ciências Farmacêuticas, Universidade de São Paulo, São Paulo, Brazil; ^4^Instituto René Rachou (IRR), Fiocruz Minas Gerais, Belo Horizonte, Brazil; ^5^Departamento de Genética, Evolução, Imunologia e Microbiologia, Instituto de Biologia, Universidade Estadual de Campinas (UNICAMP), Campinas, Brazil; ^6^Instituto de Medicina Tropical Alexander von Humboldt, Universidad Peruana Cayetano Heredia, Lima, Perú; ^7^Fundação de Medicina Tropical, Dr. Heitor Vieira Dourado (FMT-HVD), Manaus, Brazil

**Keywords:** tryptophan, kynurenine, *Plasmodium*, IFN-γ, inflammation, Indoleamine 2, 3- Dioxygenase 1

## Abstract

The human immune response that controls *Plasmodium* infection in the liver and blood stages of the parasite life cycle is composed by both pro- and anti-inflammatory programs. Pro-inflammatory responses primarily mediated by IFN-γ controls the infection, but also induce tolerogenic mechanisms to limit host damage, including the tryptophan (TRP) catabolism pathway mediated by the enzyme Indoleamine 2,3-Dioxygenase (IDO1), an enzyme that catalyzes the degradation of TRP to kynurenines (KYN). Here we assessed total serum kynurenines and cytokine dynamics in a cohort of natural *Plasmodium vivax* human infection and compared them to those of endemic healthy controls and other febrile diseases. In acute malaria, the absolute free kynurenine (KYN) serum levels and the KYN to TRP (KYN/TRP) ratio were significantly elevated in patients compared to healthy controls. Individuals with a diagnosis of a first malaria episode had higher serum KYN levels than individuals with a previous malaria episode. We observed an inverse relationship between the serum levels of IFN-γ and IL-10 in patients with a first malaria episode compared to those of subjects with previous history of malaria. Kynurenine elevation was positively correlated with serum IFN-γ levels in acute infection, whereas, it was negatively correlated with parasite load and *P. vivax* LDH levels. Overall, the differences observed between infected individuals depended on the number of *Plasmodium* infections. The decrease in the KYN/TRP ratio in malaria-experienced subjects coincided with the onset of anti-*P. vivax* IgG. These results suggest that *P. vivax* infection induces a strong anti-inflammatory program in individuals with first time malaria, which fades with ensuing protective immunity after subsequent episodes. Understanding the tolerance mechanisms involved in the initial exposure would help in defining the balance between protective and pathogenic immune responses necessary to control infection and to improve vaccination strategies.

## Introduction

Malaria is a parasitic disease that represents a significant global health problem. *Plasmodium vivax* infects over 20 million people each year (World Health Organization, 2017), and is the most geographically widespread species worldwide (Battle et al., 2019). *P. vivax* infection accounts for more than 85% of the malaria cases in Brazil, and most of the patients are confined to the Amazon region, with isolated cases occurring in other states of the country (Oliveira-ferreira et al., 2010). *P. vivax* is often present in peripheral blood at sub-patent densities and infections include a dormant liver stage invisible to current diagnostic methods, increasing the challenge for its control. Compared to *P. falciparum*, *P. vivax* infection has lower parasitic load, and disease complications are rare, but several recent studies have reinforced the association between severe disease and death in *P. vivax* infections (Poespoprodjo et al., 2009; Alexandre et al., 2012; Mahgoub et al., 2012; Baird, 2013). Overall, *Plasmodium* infections can produce severe forms of the disease, because of an insufficient immune response to control the parasite load or the inability of the host to control inflammation, resulting in immunopathology (Gonçalves et al., 2012; Mendonça and Barral-Netto, 2015), but the mechanisms involved are not fully understood (Cunnington et al., 2013; Crompton et al., 2014).

The innate immune response is pivotal for the initial control of infection and for instructing and directing the ensuing adaptive response. Clinical data from cohorts and experimental evidence from animal models supports the observation that the adaptive immune response, mainly via antibody-mediated anti-plasmodium immunity, restricts the infection and limits pathology (Longley et al., 2016). In addition, the pivotal role of IFN-γ as a central cytokine in controlling *Plasmodium* infection in both the liver and blood stages of the parasite life cycle is well documented (King and Lamb, 2015). Plasmodium infection induces IFN-γ production from a range of innate and adaptive immune cell subsets at different stages of the life cycle, highlighting the roles of both innate and adaptive immunity in controlling the infection. Although *Plasmodium*-specific antibodies are known to play a key role in controlling malarial fever and parasitemia, less is known about cellular and innate immunity to malaria in humans. *Plasmodium*-induced IFN-γ production has been related with clinical immunity to malaria in humans; yet, IFN-γ can induce pathology if not regulated (Andrade et al., 2010; Deroost et al., 2016). Therefore, the mechanisms regulating the balance between immunity and immunopathology during malaria remain unclear. Recent studies have shown that, relative to malaria-experienced individuals, naive individuals had increased activation of pro-inflammatory pathways during primary infection, despite lower parasitemia (Tran et al., 2016), providing evidence for modulation of inflammatory responses during malaria.

The IFN-γ pathway is both an important effector of innate cell-intrinsic immunity, and a key inducer of appropriate subsequent T and B cell adaptive responses. It initiates both potent pro- and anti-inflammatory cell-intrinsic responses in immune cells and in tissue, and their balance and timing can affect the ensuing adaptive mechanisms engaged to control the infection. In addition, IFN-γ strongly induces the activation of the tryptophan degradation pathway, mediated by IDO1, and a large body of evidence indicates that the tryptophan catabolism pathway is involved directly or indirectly in the host response to infection. IDO1, IDO2, and TDO catalyze the first rate-limiting step in this pathway, producing both tryptophan depletion and tryptophan catabolic products collectively known as kynurenines. Both, tryptophan depletion and kynurenines production, can convert naïve T cells into regulatory T cells. In addition, L-kynurenine, the main degradation product, activates the aryl hydrocarbon receptor (AhR), with immunoregulatory effects in both T and myeloid cells (Fallarino et al., 2006; Mellor and Munn, 2008; Grohmann and Puccetti, 2015; Yeung et al., 2015). The consequences of the activation of the tryptophan degradation pathway in chronic viral and bacterial infections, and in intracellular parasite disease, have demonstrated that both, its antimicrobial and tolerogenic effects, can influence the outcome of infection (Munn and Mellor, 2013; Schmidt and Schultze, 2014; Yeung et al., 2015).

We therefore aimed to explore the dynamics of the potent IFN-γ-induced cell intrinsic response mediated by the tryptophan catabolic pathway, assessing its potential role during the development of anti-*P. vivax* malaria immunity in an endemic region. We also aimed to explore its potential role for immunomonitoring disease dynamics in the context of natural infection, where multiple exposures to *P. vivax* are prevalent and define natural protection to the parasite.

## Materials and Methods

### Ethics Statement

This study was approved by the Universidade Federal do Amazonas (UFAM). All the study participants provided an informed consent prior to enrollment.

### Study Design

All study participants were recruited at the Fundação de Medicina Tropical Dr. Heitor Vieira Dourado (FMT-HVD; Manaus, Amazonas state) from March 2015 to February 2016. We analyzed the immune response to natural *P. vivax* infection by measuring different hematological and immune parameters (serum cytokines, tryptophan, and kynurenines levels) in an endemic region.

**FIGURE 1:**
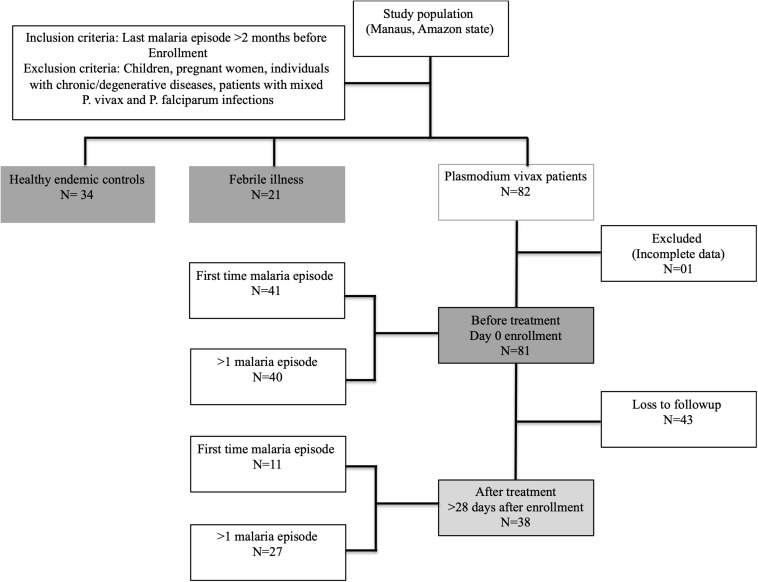
Sample collection flow chart (Strobe flow chart).

A cross-sectional study included three study groups: malaria cases (*P. vivax*-diagnosed patients, with either a first-time malaria episode or more than one episode, *n* = 81), patients with other febrile diseases (non-malaria disease, *n* = 21), and healthy malaria-negative endemic controls (*n* = 34) (Figure 1). The clinical spectrum of malaria in the participants ranged from a very mild illness to full-blown paroxysms, and none of the patients had severe or complicated disease. Subjects with a primary malaria episode were defined as subjects with a self-reported history of no previous malaria and seronegative for MSP-1_19_ *P. vivax* antigen. Subjects with previous malaria episodes were identified as subjects that self-reported previous malaria and were seropositive for IgG MSP-1_19_; last malaria episode was at least 2 months before inclusion in the study. One malaria patient was excluded from the analyses due to missing information. Febrile disease patients were confirmed as negative for *P. vivax* infection by thick blood smear microscopy. Healthy endemic controls had negative *P. vivax* diagnosis by thick blood smear microscopy and were also seronegative for malaria, identified by detection of IgM and IgG antibodies against MSP-1_19_ antigen. Individuals with chronic/degenerative diseases, pregnant women or subjects <18 years old were not enrolled in this study.

The study was exploratory and the sample size was not pre-defined by effect size, but limited by voluntary participation and enrollment. Previous malaria history and the number of days with fever and other symptoms before diagnosis were assessed by self-reported medical history. The degree of pre-existing immunity was assessed by the presence of serum anti-*P. vivax* antibodies.

A follow-up assessment was performed to evaluate the dynamics of the hematological and immune response parameters in *P. vivax*-positive patients after successful anti-malaria treatment and clearance of blood-stage parasites, as recommended by the Brazilian Ministry of Health. However, only 47% of the recruited patients returned for followup after anti-malaria treatment [before treatment (BRx, *n* = 81), and after treatment (ARx, *n* = 38), respectively]. After treatment, all patients were malaria-negative, confirmed by thick blood smear microscopy.

### Peripheral Blood Samples

Peripheral blood samples were obtained from 136 participants. A total of 5 mL of blood was drawn from each participant (using EDTA tubes, BD Vacutainer) and divided into two parts: one part was used for hematological analysis and a second part was used for blood plasma analysis. Whole blood samples were employed for assessing hematological parameters using an automated hematological analyzer (Sysmex KX-21 N^®^): Hematocrit, Hemoglobin, White blood cells, Red blood cells, Mean corpuscular volume, Mean corpuscular hemoglobin, Mean corpuscular hemoglobin concentration, Mean platelet volume, Platelets.

### Tryptophan and Kynurenine Quantification

After separation from whole blood, plasma samples (200 uL) were treated with 8% Perchloric acid, to precipitate proteins and extract soluble metabolites. After extraction, the aqueous phase was analyzed by High Performance Liquid Chromatography (HPLC) as previously described (Mallmann et al., 2018). For the standard curve, a serial dilution was performed with TRP and KYN (TRP/KYN μM/L): 100/10; 50/5; 25/2.5; 12.5/1.25; 6.25/0.625; 3.125/0.325. HPLC flow rate was 1.0 mL min^–1^ and 20 μL of clear sample was then injected into the HPLC system using an autosampler. TRP was identified at 278 nm and KYN at 360 nm by UV detection (Zhang et al., 2009). Retention time (Rt) was used to identify metabolites in the chromatogram and standard curve was constructed by plotting the ratio of peak area (computed by LCsolution software, Shimadzu) of TRP or KYN (*y*) against known TRP or KYN concentration (*x*), respectively. The linearity of the standard curves was confirmed using regression variance analysis and significance of correlation co-efficient (*r*) checked using Student’s *t*-test. KYN/TRP ratio was calculated using the formula KYN concentration (μM/L)/TRP concentration (μM/L).

### Cytokines Quantification

Plasma cytokines (IL-2, IL-4, IL-6, IL-10, TNF, IFN-γ, and IL-17A) levels were quantified using the CBA Th1, Th2, and Th17 kit (Cytometric Bead Array, BD Biosciences), following the manufacturer’s instructions. Samples were analyzed on a FACSCanto II (Becton, Dickinson and Company, San Jose, CA, United States) flow cytometer and data analyzed by FCAP ArrayTM software (V3.0.1). The mean fluorescence intensity (MFI) of each bead cluster was determined and forth logistic regression applied to build the standard curves. Cytokine concentrations for each sample were then extrapolated from the standard curves and data was expressed as pg/mL for each plasmatic cytokine.

### Lactate Dehydrogenase (LDH) ELISA

Plasma samples were analyzed by an *in-house* sandwich ELISA to detect *P. vivax* LDH (PvLDH) in untreated malaria patients as previously described (Sousa et al., 2014). Briefly, ELISA plates were coated with polyclonal anti-PvLDH rabbit antibodies, next patient plasma samples were added to capture cell-free PvLDH. Captured antigen was then identified using a mouse PvLDH-specific primay antibody and a HRP-conjugated goat anti-mouse IgG secondary antibody. Absorbance cut-offs were calculated to determine reactive samples using the mean optical density readings from negative sample plus twice the standard deviation of negative samples.

### *Plasmodium vivax* Serology

Plasma samples were analyzed by indirect ELISA to detect *P. vivax-*specific total IgG and IgM antibodies using the 19-kDa C-terminal region of the Merozoite Surface Protein-1 of P. vivax (MSP-1_19_), as previously described (Soares and Rodrigues, 2002). Briefly, recombinant antigen was coated on an ELISA plate, and after blocking, patient plasma samples were added and incubated. Subsequently, HRP-conjugated anti-human IgG or IgM secondary antibodies were added, and optical density readings recorded. For each sample, the reactivity index (RI: absorbance/cut-off) was calculated.

### Statistics

Differences in means and medians were tested, respectively, using either one-way ANOVA, with Tukey’s *post hoc* test, or the Mann-Whitney *U* test or Kruskal-Wallis test, with Dunn’s *post hoc* test, with correction for multiple comparisons as appropriate. The correlation between variables was determined using the Pearson’s correlation coefficient. Data was analyzed using GraphPad Prism version 6 (GraphPad Software, La Jolla, CA, United States) and Rstudio version 1.1.4 with R version 3.5.0 and RPQ, HNP and Tidyverse packages.

## Results

### Demographic Description of the Study Groups

A total of one hundred and thirty-six individuals were recruited for this study. One patient was excluded (because of missing information), and out of eighty-one malaria patients, forty-three patients were lost to follow-up after treatment (Figure 1). Table 1 and Supplementary Table S1 summarize the demographic data and hematological results of the study groups: (1) healthy endemic controls, (2) individuals with non-malarial febrile illness, (3) *P. vivax* malaria patients (enrolled on Day 0 before administering anti-malarial treatment), and (4) *P. vivax* malaria patients followed-up after successful anti-malarial treatment. Median (Inter quartile range, IQR) parasite load/mm^3^, estimated by thick blood smear, was 2158 (537.6−4185) in *P. vivax* malaria patients, and the mean parasite load was not different between patients with a first-time malaria episode, and individuals with previous malaria history [>1 malaria episode, median episode number = 3 (IQR: 2-5.75)] (Figure 3B).

**TABLE 1:** Patient clinical and demographic details.

Demographic characteristics		Healthy endemic	Febrile patients	*Plasmodium vivax*		**p*-value^AB^*	**p*-value^BC^*	**p*-value^AC^*	**p*-value^CD^*
				
		(*n* = 34)^A^	(*n* = 21)^B^	Before malaria treatment (*n* = 81)^#*C*^	After malaria treatment (*n* = 38)^D^				
Gender	Female (%)	12 (35%)	10 (48%)	22 (27%)	6 (15%)				
Age, mean	Years ± SD	34.7 ± 11.7	40.3 ± 14.6	38.7 ± 10.8	39.9 ± 10.6				
Number of malaria episodes	First malaria			41 (51%)	11 (31%)				
	>1 malaria			40 (49%)	27 (69%)				
Days with fever (symptoms)§	<6 days		11 (73%)	52 (69%)	24 (65%)				
	>6 days		4 (27%)	23 (31%)	13 (35%)				
**Hematological parameters (mean ± SD)**									
Hematocrit (%)		45.9 ± 5.0	40.5 ± 2.8	41.9 ± 6.7	44.9 ± 3.3	0.0006	> 0.9999	0.0002	<0.0005
Hemoglobin (g/dL)		16.4 ± 12.2	13.9 ± 0.9	13.8 ± 2.3	13.8 ± 1.3	> 0.9999	> 0.9999	0.1852	> 0.9999
White blood cells (×10^3^/μL)		6.4 ± 1.1	5.7 ± 1.4	4.5 ± 1.8	5.9 ± 1.4	0.0627	0.3913	<0.0001	<0.0001
Red blood cells (×10^6^/μL)		5.2 ± 6.4	4.2 ± 1.3	5.1 ± 8.7	4.9 ± 5.1	0.0005	0.0038	0.6103	> 0.9999
Mean corpuscular volume (fL)		89.3 ± 4.5	89.4 ± 5.2	87.2 ± 3.4	89.3 ± 3.2	> 0.9999	0.2684	0.0398	0.0081
Mean corpuscular hemoglobin (pg)		27.6 ± 1.4	30.7 ± 2.1	27.9 ± 2.4	27.8 ± 1.4	0.0004	<0.0001	> 0.9999	> 0.9999
Mean corpuscular hemoglobin concentration (g/dL)		31.1 ± 0.9	34.3 ± 1.1	31.6 ± 1.6	31.1 ± 0.8	<0.0001	<0.0001	0.2776	0.8746
Mean platelet volume (fL)		9.3 ± 0.9	8.5 ± 0.8	10.5 ± 1.1	9.8 ± 1.1	0.6782	<0.0001	0.0011	0.1037
Platelet (×10^3^/μL)		256 ± 71.6	246 ± 62	110 ± 57.3	235 ± 56.9	> 0.9999	<0.0001	0.0001	0.0001

*Before malaria treatment (BRx): Day 0 on enrolment; After malaria treatment (ARx):>28 days after treatment; >1 malaria episodes, Median episode number = 3 (IQR: 2-5.75)]; ^#^Median Parasitemia (Parasites/mm^3^) = 2158 (IQR, 537.6 - 4185); §Missing patient data.*

**FIGURE 2:**
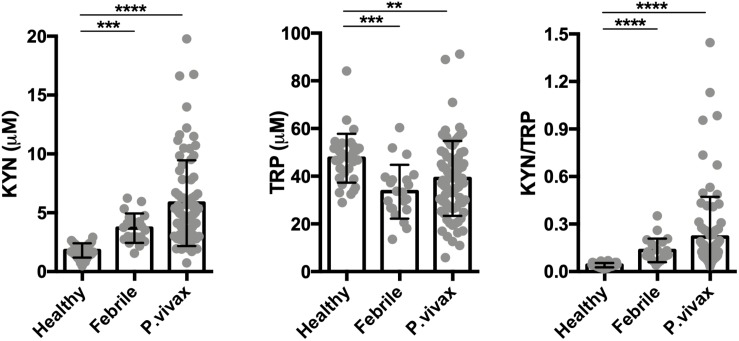
The tryptophan degradation pathway is activated in acute infection. Kynurenine (KYN), tryptophan (TRP) levels were quantified in healthy endemic control group (*n* = 34), non-malarial febrile patients (*n* = 21) and vivax infected patients before commencing anti-malarial treatment (*n* = 81) by HPLC. (**P < 0.01, ***P < 0.001, ****P < 0.0001, ANOVA test with Tukey’s *post hoc* test).

**FIGURE 3:**
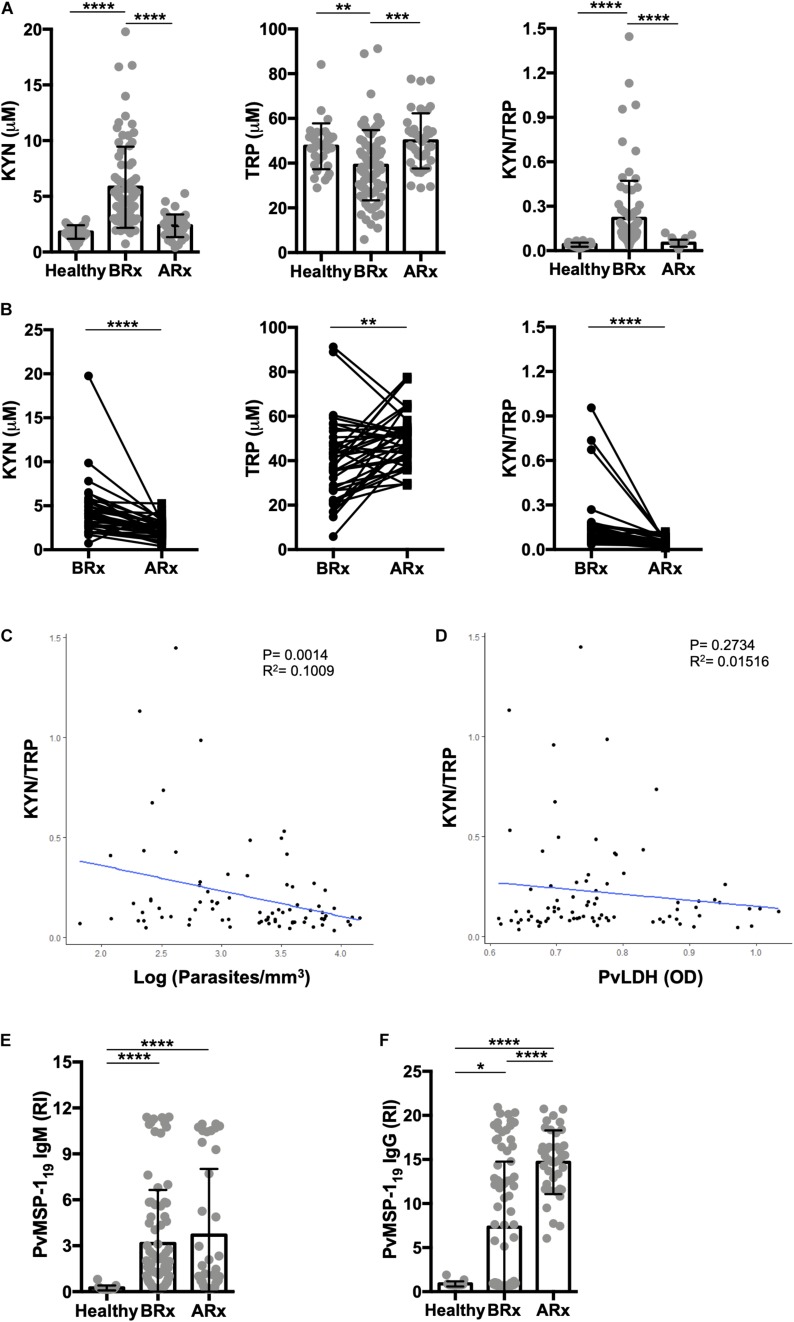
Successful antimalarial treatment reverts kynurenine levels. **(A)** Serum kynurenine (KYN) and tryptophan (TRP) levels were quantified by HPLC in a healthy endemic control group (*n* = 34), and in *P. vivax*-infected patients on day 0 of enrollment (before starting anti-malarial treatment, BRx, *n* = 81), and >28 days after successful malaria treatment (ARx, *n* = 38). **(B)** Paired sample analysis before and after vivax malaria treatment (*n* = 38). Correlation analysis between KYN/TRP ratio and **(C)** parasitemia levels, quantified by thick blood film, or **(D)** vivax lactate dehydrogenase (PvLDH), measured by ELISA. *P.*vivax MSP-1_19_ antigen specific IgM **(E)** and IgG **(F)** antibodies were measured by enzyme-linked immunosorbent assay (ELISA) to confirm sero-conversion (*P < 0.05, **P < 0.01, ***P < 0.001, ****P < 0.0001, ANOVA test with Tukey’s *post hoc* test, student’s paired *t*-test and Pearson’s correlation).

### Natural *P. vivax* Infection Induces Hematological Changes, a Complex Serum Cytokine Response, and an Increased Activity of the Tryptophan Degradation Pathway

In this cross-sectional study we evaluated healthy subjects (*n* = 34), *P. vivax*-malaria patients (*n* = 81), and patients with other febrile diseases (*n* = 21). The hematological parameters and serum cytokines levels of these groups are summarized in Tables 1 and 2. We observed that malaria induced significant hematological changes that comprised a reduction in hematocrit, leukocyte, and platelet levels (Supplementary Tables S1, S2 and Supplementary Figure S1), as well as a mixed cytokine profile that included the concomitant elevation of canonical Th1, Th2, and Th17 cytokines, with a distinct elevation in serum IFN-γ and IL-10 compared to healthy controls (Table 2). These results indicate that acute *P. vivax* infection induces a significant decrease in multiple circulating blood cell lineages, together with a distinct pro- and anti-inflammatory serum cytokine response.

**TABLE 2:** Blood cytokine levels in *Plasmodium vivax* infected patients.

Cytokines pg/mL (mean ± SD)	Healthy endemic	*Plasmodium vivax*		Number of infections, patients before anti-malarial treatment		*p*-value^AB^	*p*-value^BC^	*p*-value^AC^	*p*-value^DE^
			
	(*n* = 34)^A^	Before malaria treatment (*n* = 81)^#*B*^	After malaria treatment (*n* = 38)^C^	First malaria episode (*n* = 41)^D^	>1 malaria episode§ (*n* = 40)^E^				
IL-2	0.1 ± 0.1	0.1 ± 0.4	0.1 ± 0.1	0.1 ± 0.25	0.1 ± 0.47	0.3303	0.0890	> 0.9999	0.5689
IL-4	0.4 ± 1.5	1.1 ± 4.3	0.1 ± 0.1	0.3 ± 0.78	1.7 ± 6.1	0.0056	<0.0001	> 0.9999	0.6878
IL-6	0.9 ± 1.6	104.2 ± 321.2	1.1 ± 3.2	59.1 ± 133.8	150.4 ± 434.8	<0.0001	<0.0001	> 0.9999	0.3808
IL-10	0.4 ± 1.4	304.3 ± 855.1	0.3 ± 0.8	198.3 ± 698.2	413.0 ± 988.3	<0.0001	<0.0001	> 0.9999	0.6206
IL-17	2.2 ± 5.5	23.1 ± 44.1	44.1 ± 58.4	24.3 ± 48.1	21.9 ± 40.2	0.0259	0.0102	<0.0001	0.9107
IFN-β	0.1 ± 0.1	282.4 ± 849.4	0.0 ± 0.0	533.6 ± 1153	24.2 ± 42.4	<0.0001	<0.0001	> 0.9999	0.0003
TNF-α	0.3 ± 1.3	0.42 ± 1.7	0.0 ± 0.0	0.1 ± 0.3	0.8 ± 2.3	0.7658	0.0561	> 0.9999	0.0201

*Before malaria treatment (BRx): Day 0 on enrollment; After malaria treatment (ARx): >28 days after treatment; § > 1 malaria episodes, Median episode number = 3 (IQR: 2-5.75)]; ^#^Median Parasitemia (Parasites/mm^3^) = 2158 (IQR, 537.6 - 4185).*

We assessed the activity of the tryptophan catabolism pathway by measuring kynurenines, tryptophan, and the KYN/TRP ratio in sera, a marker for overall tryptophan catabolism by the kynurenine pathway (Widner et al., 1997; Yeung et al., 2015). We observed a significant increase in both serum KYN levels and in the KYN/TRP ratio in acute disease at recruitment, before starting anti-malaria treatment (Figure 2). We also observed that other febrile diseases also induce an increase in the serum KYN and the KYN/TRP ratio compared to healthy controls. These results indicate that the induction of the tryptophan degradation pathway is a non-specific host response to infecting pathogens, as observed by others (Schmidt and Schultze, 2014; Yeung et al., 2015).

Next, we evaluated the dynamics of hematologic parameters, serum cytokines, and the tryptophan catabolism pathway in the *P. vivax*-malaria patients before and after anti-malarial chemotherapy in a subgroup that returned for evaluation. At follow up, all patients cleared the parasite after treatment (*n* = 38). All measured hematological parameters returned to levels comparable to those of the healthy controls (Table 1). In a similar fashion, serum cytokine levels returned to levels comparable to those of the healthy controls with the exception of IL-17 (Table 2). We also observed that the tryptophan degradation pathway activity (measured by the KYN/TRP ratio) returned to levels comparable to those of the control group (Figure 3).

These results show that natural *P. vivax infection* induces a reduction in circulating blood cells, a marked mixed cytokine response, and an increase in the production of tryptophan degradation products that return to healthy control levels after treatment, as seen by others and in experimental infections (Schmidt and Schultze, 2014; Yeung et al., 2015).

### A Single, First Malaria Episode Induces Higher Tryptophan Degradation Pathway Activity Compared to Multiple Infection Episodes

**FIGURE 4:**
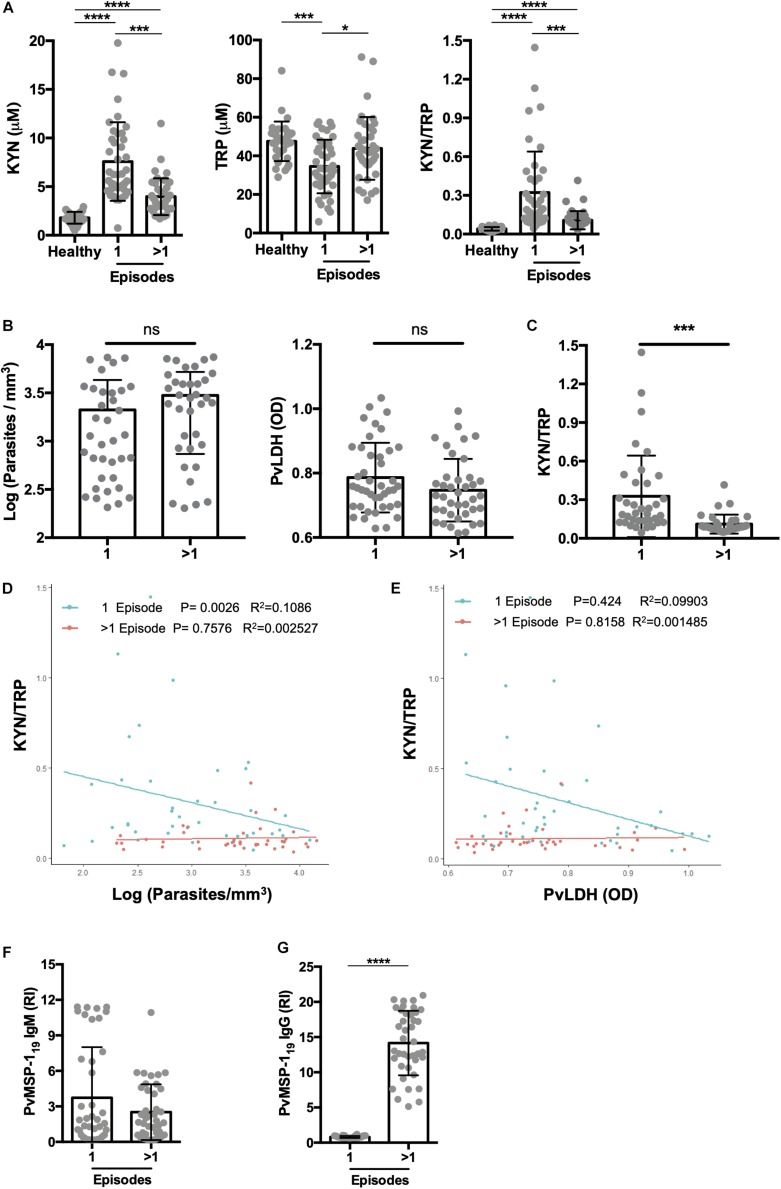
Primary malaria significantly elevates kynurenine levels. **(A)** Kynurenine (KYN) and tryptophan (TRP) levels were quantified by HPLC in healthy endemic control group, malaria-naïve or first malaria episode (1 episode), and individuals with previous malaria history (>1 *P. vivax* malaria episode), before malaria treatment. Individuals with a first malaria (*n* = 35) and previous malaria exposure (*n* = 35) were paired by panel **(B)** blood parasite levels or *P.vivax* LDH levels and **(C)** KYN/TRP ratio was compared. Correlation analysis between **(D)** parasites levels quantified by thick blood film or **(E)** *P.vivax* LDH measured by ELISA and KYN/TRP ratio, grouped by patients with a first episode (1 episode, green) or >1 previous malaria episode (>1 episode, red). **(F,G)** *P.vivax* serology was performed using MSP-1_19_ antigen to confirm malaria exposure. (*P < 0.05, **P < 0.01, ***P < 0.001, ****P < 0.0001, student’s *t*-test, ANOVA test with Tukey’s *post hoc* test and Pearson’s correlation).

**FIGURE 5:**
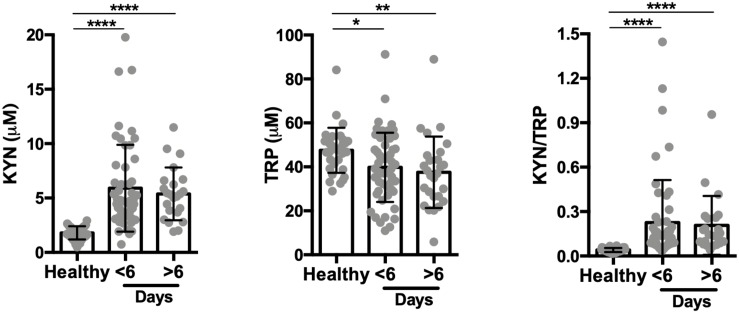
Kynurenine levels are independent of the number of febrile days before treatment. Kynurenine (KYN) and tryptophan (TRP) levels were quantified by HPLC in the healthy control group (*n* = 34) and the *P. vivax*-infected patients, before malaria treatment (*n* = 75; six patients failed to respond number of febrile days on inclusion). Data stratified by number of days with fever as self-reported by patients. (*P < 0.05, **P < 0.01, ****P < 0.0001, ANOVA test with Tukey’s *post hoc* test).

Numerous studies have shown that multiple malaria episodes can induce parasite tolerance by the host. Therefore, we next compared the relationship between the number of malaria episodes and the KYN/TRP ratio, and observed that it was elevated in subjects with a “primary” or first malaria, compared to individuals with previous malaria episodes (>1 malaria episode; Figure 4A and Supplementary Figure 2A). Moreover, no differences were observed in parasite load and *P. vivax* LDH levels of individuals with a first malaria-infection compared to the ones of individuals with a previous malaria exposure (Figures 4B,C). Interestingly, the increased elevation in the KYN/TRP ratio observed in first *Plasmodium* infection negatively correlated with parasite load, while it was independent of parasite load in patients that had a previous infection (Figures 4D,E). In addition, we observed that the IgM antibody levels were similar between first malaria patients compared to patients with a previous malaria episode (Figure 4F). As expected, first malaria-infected individuals were IgG-negative for MSP-1_19_, whereas individuals with previous malaria history had circulating antibodies (Figure 4G). We hypothesized that the number of febrile days, a proxy for systemic inflammation, would affect the activity of the tryptophan degradation pathway, however, no significant difference in kynurenine levels was observed between the patient groups when stratified by number of days with fever before anti-malaria treatment (Figure 5 and Supplementary Figure S2B). Additionally, we did not observe any relationship between blood parasite stages and KYN/TRP ratio (Supplementary Figure S3).

**FIGURE 6:**
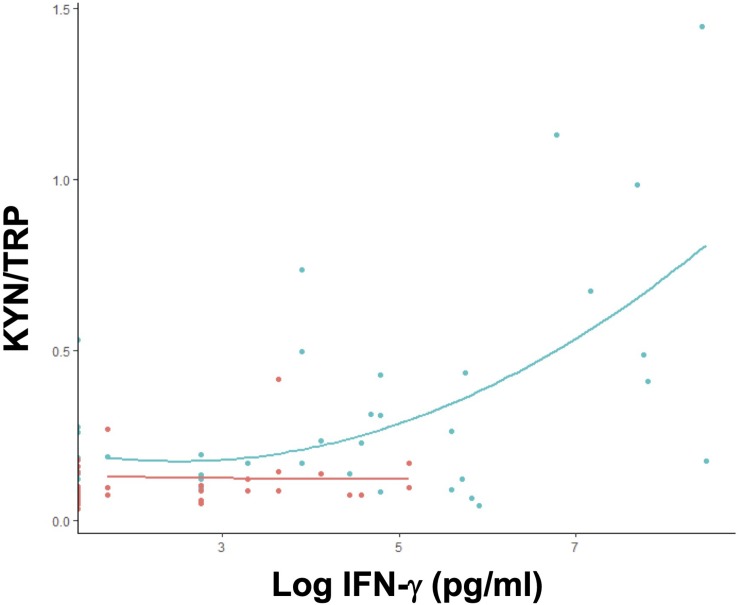
IFN-γ positively modulates tryptophan catabolism in primary *Plasmodium* infection. Correlation between plasma KYN/TRP ratio and IFN-γ levels in untreated *P. vivax* malaria patients, grouped by the number of infections. Single (1 episode, green; *n* = 41) or multiple malaria (>1 episode, red; *n* = 40) (Supplementary Table S2).

Although all cytokines were elevated in individuals with malaria compared to healthy controls, only IFN-γ and TNF-α were significantly elevated in patients with a first malaria episode compared to individuals with multiple episodes (Table 2). IL-10 was elevated in individuals with multiple infections compared to ones with first time malaria; however, the elevation was not significant (Table 2). As anticipated, elevated IFN-γ levels were associated with an increase in the KYN/TRP ratio, whereas IL-10 was negatively correlated (Supplementary Figures S1C,D). Only individuals with a first malaria episode demonstrated significant positive correlation with IFN-γ and KYN/TRP ratio (Figure 6).

These results suggest that a first episode of *P. vivax* malaria produces a distinct host response with a significant induction of the tryptophan degradation pathway, together with a higher pro-inflammatory cytokine response, which correlates positively with serum IFN-γ levels and negatively with parasitemia. In contrast, multiple infections induce a lower activity of the tryptophan degradation pathway, which is independent of parasitemia and coincides with the onset of the production of specific anti-*P. vivax* antibodies.

## Discussion

Interferon-gamma is secreted by both innate and adaptive immune cells and is essential for controlling both liver and blood stages of the parasite (King and Lamb, 2015). In addition, IFN-γ drives tryptophan catabolism by inducing the production and enzymatic activity of IDO1. The catabolites -kynurenines- mediate immune tolerance and interfere with pathogen clearance (Mellor and Munn, 2008; Schmidt and Schultze, 2014). *Plasmodium* parasites can synthesize and acquire tryptophan; therefore it is assumed that the activation of the tryptophan degradation pathway only modulates the host response (Liu et al., 2006). In this study, we observed a marked elevation of IFN-γ with a concomitant induction of the tryptophan degradation pathway measured in the serum of naive individuals with a first documented malaria episode, compared to patients with previous malaria. Interestingly, the kynurenine elevation observed in patients with a first *P. vivax* infection was negatively correlated with blood parasite load. We also observed a lack of correlation between parasitemia and level of exposure, a phenomenon described by others (Gonçalves et al., 2014). This may indicate that the potent induction of IFN-γ could be controlling parasite load and simultaneously activating the tryptophan degradation pathway via IDO1 in malaria-naïve individuals, and that in non-naïve individuals in which adaptive immunity is active, the overall response shifts toward lower IFN-γ levels and anti-malaria antibodies, and is independent of parasite load. The shift to antibody-mediated responses and lower pro-inflammatory cytokines is a well-documented phenomenon in the establishment of parasite tolerance and control (Gonçalves et al., 2012; Chaves et al., 2016; Deroost et al., 2016; Longley et al., 2016; Pires et al., 2018).

Myeloid and antigen-presenting cells, as well as parenchymal cells are important for KYN production (Mellor and Munn, 2004). Tryptophan-derived metabolites produced by these cells mediate immune tolerance by inducing apoptosis of activated T cells and by the conversion of naive T cells into T regulatory cells (Tregs), via the activation of the aryl hydrocarbon receptor; tryptophan restriction induces the starvation response in T cells via the activation of the GCN2 kinase (Terness et al., 2002; Mellor and Munn, 2008; Mezrich et al., 2010; Nguyen et al., 2010). Our observations are consistent with recent studies that characterized serum metabolomics of *Plasmodium*-infected humans and non-human primates, and controlled human malaria trials (Woodberry et al., 2017; Vallejo et al., 2018). A recent study that used a controlled human *P. vivax* infection model showed an elevation of the plasmatic KYN/TRP ratio and an increase in activated regulatory T cells (Woodberry et al., 2017), and our findings corroborate these results. Interestingly, we observed a negative correlation between the KYN/TRP ratio and parasitemia, suggesting that the innate immune response is activated at a low blood parasitemia level, compared to what is observed the clinical experimental setting (Woodberry et al., 2017). Our data from a natural infection cohort also corroborates recent published data showing an increased activity of the tryptophan degradation pathway in subjects with no previous history of malaria disease compared to subjects with history of previous exposure (Gardinassi et al., 2018; Cordy et al., 2019). We recently observed that acute *Plasmodium* infection induced an IFN-γ-driven increment in serum kynurenines that correlated with an elevation in the frequency of circulating FoxP3^+^ T regulatory cells in a hypo-endemic Amazon region (Dos Santos et al., 2020). Our exploratory results grant a more detailed prospective analysis of the relationship between innate immunity, parasitemia, and the balance of pro and anti-inflammatory pathways in human malaria and its dynamics during multiple episodic infections. Of note, we also observed that the tryptophan degradation pathway activity was increased in non-malaria disease as well, suggesting that this pathway is activated by broad non-specific host responses to acute inflammation (Cordy et al., 2019). Previous studies that evaluated the role of the tryptophan degradation pathway in malaria have consistently shown that the activity of IDO1 is deleterious, and that it is associated with the development of disease complications (cerebral malaria, severe disease, hypotension, among others) (Sanni et al., 1998; Hansen et al., 2000; Tetsutani et al., 2007; Wang et al., 2010; Woodberry et al., 2017). Although it is clear that IDO1 plays a significant role in the pathogenesis of the disease and its complications, the bulk of the data is either from *in vitro* cell studies *or in vivo* mouse models. Accordingly, more human data are needed to clearly establish the relevance of these findings.

Our understanding of how, in the context of moderate to high exposure to parasite and host components in the bloodstream of malaria-infected subjects, the majority of clinical cases lack overt clinical disease and complications is still limited (Sinton, 1938; Crompton et al., 2014). In addition to evolutionary human adaptation to malaria parasites that confer host resistance (Júnior et al., 2010; Piel et al., 2013; Vale, 2018; Wang et al., 2018), multiple mechanisms have been proposed to explain malaria tolerance. In this study, we observed that IFN-γ and serum kynurenines increase to both control the infection and to avoid host immunopathology. Our observations in a natural infection cohort suggest that the potent activation of the tryptophan degradation pathway might be part of a host response that uses inflammation to minimize infection intensity while balancing resistance and tolerance (Sears et al., 2011; Ayres and Schneider, 2012). Accordingly, recent studies have demonstrated the role of IDO1 in the induction of host tolerance to inflammation (Bessede et al., 2014; Mondanelli et al., 2017). Our data proposes that a potent IFN-γ-induced IDO1 response might provide protection to naive hosts via parasite tolerance in the context of sub-optimal adaptive immunity, while subsequent tolerated exposures improve adaptive responses via increased antigen exposure and ensuing adaptive immunity, independent of parasite load.

Different clinical outcomes during *Plasmodium* infections have been mainly attributed to differences in host immune responses and parasite load (Garver et al., 2014). These factors, together with pre-existing immunity, either natural or vaccine-induced, can modify disease progression and its complications. Vaccination strategies that explore boosting IFN-γ responses might be essential in improving disease outcome, but their effects in human immune activation function are still poorly defined. In this regard, our observations may be relevant for anti-malaria vaccination strategies, since improving IFN-γ responses via vaccination will increase the activity of the tryptophan degradation pathway. In a recent tuberculosis vaccine trial that failed to show protection, baseline IDO activity negatively correlated with vaccine-specific IFN-γ responses, suggesting that IDO1 activity may impair the generation of T cell memory responses (Tanner et al., 2014). Our study was limited by several factors, including a relatively low number of participants, and the lack of a baseline metabolic assessment before the infecting episodes. We also observed a ∼50% of participant dropout in the study follow-up, limiting the interpretation of our follow-up preliminary findings. We also acknowledge that the measurement of the TRP/KYN ratio is a crude approximation (or proxy) of the activity of the inducible tryptophan degradation pathway, since multiple factors, including diet and other concomitant infections can have an effect on serum TRP or KYN levels (Yeung et al., 2015). Our exploratory results grant a more detailed assessment of the tryptophan degradation pathway in a natural infection setting.

## Conclusion

We observed that in the setting of natural infection, a first *P. vivax* infection produces an increased activity of the IFN-γ-driven tryptophan catabolism pathway, an effect that fades after subsequent exposures. Since IFN-γ-induced tryptophan degradation is a potent and conserved host response, exploring malaria disease tolerance mechanisms induced via tryptophan-derived catabolites can be important for understanding the balance between protective and pathogenic immune responses to the parasite, and may help further vaccination strategies and in the design and interpretation of controlled human infection studies.

## Data Availability Statement

All datasets generated for this study are included in the article/Supplementary Material.

## Ethics Statement

The studies involving human participants were reviewed and approved by Universidade Federal do Amazonas (UFAM). The patients or participants provided their written informed consent to participate in this study.

## Author Contributions

RS, ML, and PL contributed conception and study design. RS, MC, and SL collected and analyzed the patient samples. LO, PN, IS, and FK provided reagents and performed malaria serology. EL assisted with HPLC analysis. AC provided reagents and cytokine analysis. RS, FC, CG, and PL analyzed the data and prepared the figures. RS, CG, and PL wrote the manuscript. All authors contributed to manuscript revision, read and approved the submitted version.

## Conflict of Interest

The authors declare that the research was conducted in the absence of any commercial or financial relationships that could be construed as a potential conflict of interest.

## Supplementary Material

FIGURE S1 Correlation between platelets, leukocytes cytokines and KYN/TRP ratio. Correlation between **(A)** platelets, **(B)** leukocytes, **(C,D)** cytokines and KYN/TRP ratio in malaria-infected patients before treatment (*n* = 81) was evaluated (Pearson’s correlation).

FIGURE S2 The tryptophan degradation pathway is elevated in acute vivax infection, decreases with subsequent malaria episodes, and is independent of the number of days with fever before treatment. Kynurenine (KYN) and tryptophan (TRP) levels were quantified in healthy control group (*n* = 34) and vivax infected patients before malaria treatment by HPLC. Data was then stratified by panel **(A)** number of malaria episodes or **(B)** number of days with fever before enrollment as self-reported by patients. (^∗^*P* < 0.05, ^∗∗^*P* < 0.01, ^****^*P* < 0.0001, Kruskal-Wallis test with Dunn’s *post hoc* test).

FIGURE S3 Vivax malaria blood stage compared with KYN/TRP ratio. At least hundred parasites or fifty microscopic fields were counted to estimate percentage of parasite blood stages as observed in thick smear stained with Hematoxylin and Eosin. Each column represents one vivax positive patient.

TABLE S1 Hematological characteristic of patients with *Plasmodium vivax* infection.

TABLE S2 Estimates of parameters based on gamma regression model for KYN/TRP ratio and Interferon-gamma.
