# Supplementary figures and images for: A First Plasmodium vivax Natural Infection Induces Increased Activity of the Interferon Gamma-Driven Tryptophan Catabolism Pathway
Source: Front Microbiol. 2020 Mar 17;11:400. doi: 10.3389/fmicb.2020.00400 (PMC7089964; doi:10.3389/fmicb.2020.00400)

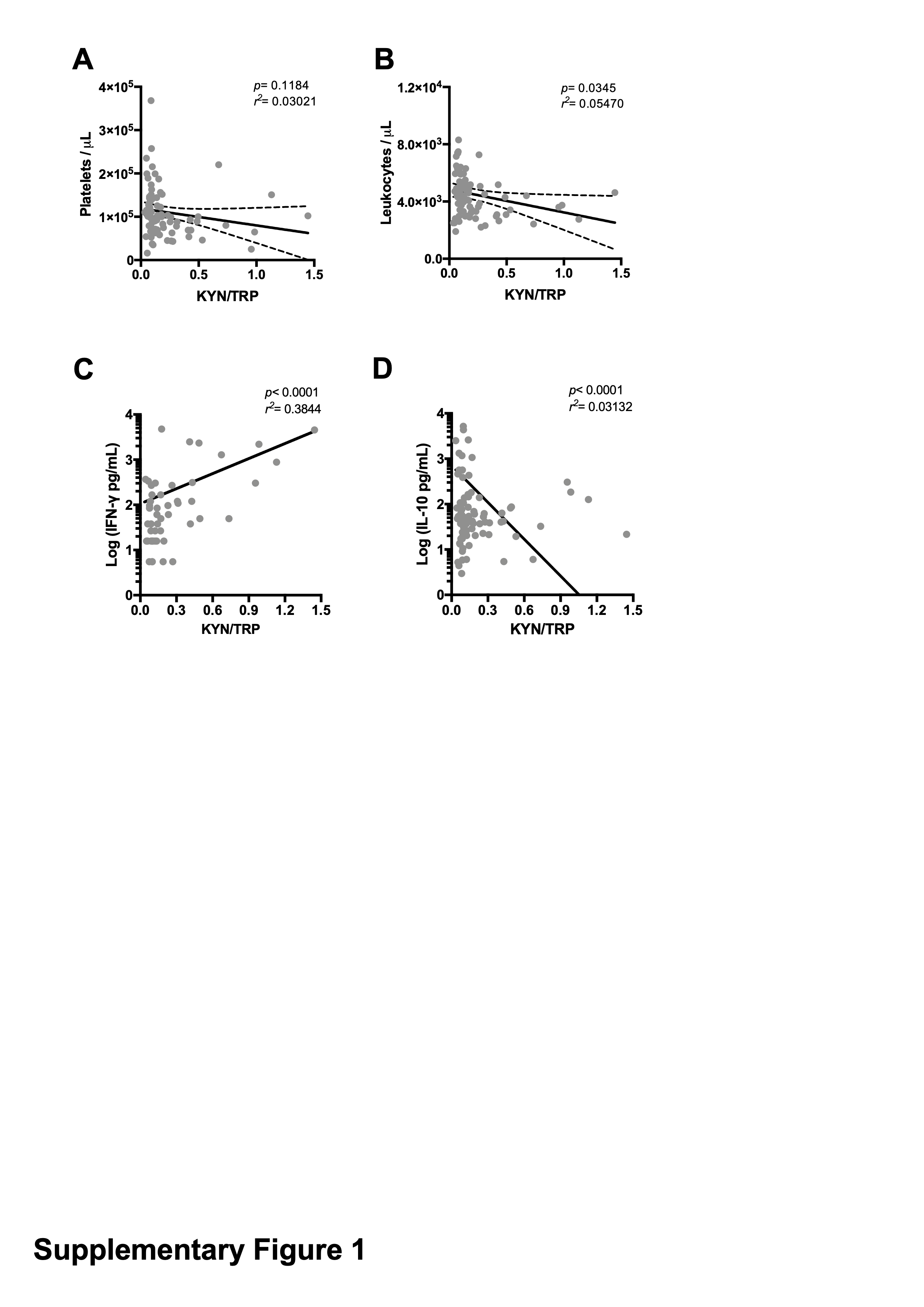

Supplement: FIGURE S1 — Correlation between platelets, leukocytes cytokines and KYN/TRP ratio. Correlation between (A) platelets, (B) leukocytes, (C,D) cytokines and KYN/TRP ratio in malaria-infected patients before treatment (n = 81) was evaluated (Pearson’s correlation). [file Image_1.TIFF]
