# Supplementary material for: A First Plasmodium vivax Natural Infection Induces Increased Activity of the Interferon Gamma-Driven Tryptophan Catabolism Pathway
Source: Front Microbiol. 2020 Mar 17;11:400. doi: 10.3389/fmicb.2020.00400 (PMC7089964; doi:10.3389/fmicb.2020.00400)
Supplement: TABLE S1 — Hematological characteristic of patients with Plasmodium vivax infection. [file Table_1.DOCX]

**Supplementary Table 1: Haematological characteristic of patients with malaria infection**

| **Haematological parameters**  **(mean ± SD)** | **Healthy** | **First time malaria** | | **>1 malaria infection** | | ***p value^AB^*** | ***p value ^AD^*** | ***p value ^BD^*** | ***p value ^AC^*** | ***p value ^AE^*** |
| --- | --- | --- | --- | --- | --- | --- | --- | --- | --- | --- |
|  | **(n=34)^A^** | **Before malaria treatment (n=41)^B^** | **After malaria treatment (n=11)^C^** | **Before malaria treatment (n=40)^D^** | **After malaria treatment (n=27)^E^** |  |  |  |  |  |
| HCT (%) | 45.9 ± 5.0 | 40.9 ± 5.3 | 44.5 ± 4.1 | 42.9 ± 7.9 | 45.23 ± 3.1 | **0.0001** | >0.9999 | 0.5351 | >0.9999 | >0.9999 |
| HGB g/dL | 16.4 ± 12.2 | 13.8 ± 2.1 | 13.7 ± 1.5 | 13.8 ± 2.5 | 13.9 ± 1.3 | >0.9999 | >0.9999 | >0.9999 | >0.9999 | >0.9999 |
| WBC (unid. x 103/µL) | 6.4 ± 1.1 | 3.9 ± 1.2 | 5.4 ± 1.4 | 5.1 ± 2.1 | 6.1 ± 1.4 | **<0.0001** | **0.0005** | **0.0359** | 0.3666 | >0.9999 |
| RBC (unid. x 106/µL) | 5.2 ± 6.4 | 4.9 ± 8.4 | 5.1 ± 5.7 | 5.1 ± 9.1 | 4.9 ± 4.9 | >0.9999 | >0.9999 | >0.9999 | >0.9999 | >0.9999 |
| MCV (fL) | 89.3 ± 4.5 | 86.1 ± 2.3 | 83.7 ± 4.3 | 88.1 ± 2.9 | 88.2 ± 2.9 | 0.1522 | >0.9999 | >0.9999 | **0.0385** | >0.9999 |
| MCH (pg) | 27.6 ± 1.4 | 28.4 ± 3.5 | 27.3 ± 2.1 | 27.7 ± 1.4 | 27.8 ± 1.4 | >0.9999 | >0.9999 | >0.9999 | >0.9999 | >0.9999 |
| MCHC (g/dL) | 31.1 ± 0.9 | 32.4 ± 2.5 | 32.4 ± 1.5 | 31.2 ± 0.7 | 31.2 ± 0.7 | 0.1212 | >0.9999 | >0.9999 | 0.1094 | >0.9999 |
| MPV (fL) | 9.3 ± 0.9 | 10.6 ± 1.3 | 10.3 ± 1.3 | 10.7 ± 0.9 | 10.7 ± 0.9 | **0.0275** | **0.0235** | >0.9999 | 0.8055 | **0.0235** |
| PLT (unid. x 103/µL) | 256 ± 71.6 | 96 ± 46.1 | 227 ± 61.6 | 127± 62.8 | 236 ± 56.8 | **<0.0001** | **<0.0001** | 0.7873 | >0.9999 | >0.9999 |

Before malaria treatment (BRx): Day 0 on enrolment; After malaria treatment (ARx): >28 days after treatment; >1 malaria episodes, Median episode number= 3 (IQR: 2-5.75)]; Haematocrit (%); Haemoglobin (g/dL); White blood cells (x 103/µL); Red blood cells (x 106/µL); Mean corpuscular volume (fL); Mean corpuscular haemoglobin (pg); Mean corpuscular haemoglobin concentration (g/dL); Mean platelet volume (fL); Platelet (x 10^3^/µL).
