# Supplementary material for: A First Plasmodium vivax Natural Infection Induces Increased Activity of the Interferon Gamma-Driven Tryptophan Catabolism Pathway
Source: Front Microbiol. 2020 Mar 17;11:400. doi: 10.3389/fmicb.2020.00400 (PMC7089964; doi:10.3389/fmicb.2020.00400)
Supplement: TABLE S2 — Estimates of parameters based on gamma regression model for KYN/TRP ratio and Interferon-gamma. [file Table_2.docx]

**Supplementary Table 2: Estimates of parameters based on gamma regression model for KYN/TRP ratio and Interferon-gamma**

| **Coefficients** | **Estimate** | **Std. Error** | **p value** |
| --- | --- | --- | --- |
| (Intercept) | -2.77542 | 0.28414 | **1.08e-12 ***** |
| Log (IFN-γ) | 0.21227 | 0.06562 | **0.00228**** |
| Infection episodes | 0.54071 | 0.24472 | **0.03227*** |
